# The Course of Parental Psychological Distress in Childhood and Adolescent Depression

**DOI:** 10.1016/j.jaacop.2024.05.003

**Published:** 2024-07-08

**Authors:** Katia Mace, Maria Sifaki, Emily Midouhas, Eirini Flouri, Efstathios Papachristou

**Affiliations:** a UCL Institute of Education, London, United Kingdom; b UCL Institute of Epidemiology and Health Care, London, United Kingdom

**Keywords:** adolescence, depression, maternal psychological distress, millennium cohort study, paternal psychological distress

## Abstract

**Objective:**

Investigations of the influence of parental psychological distress on depression in offspring have largely focused on maternal, rather than paternal, symptoms. This study examined how psychological distress trajectories of both fathers and mothers across their child’s preschool and primary school years relate to depressive symptoms of the child in adolescence. The aim was to assess whether maternal and paternal psychological distress symptoms develop in parallel during the childhood years and how each parent’s symptoms may influence their adolescent’s symptoms.

**Method:**

The sample comprised 8,888 Millennium Cohort Study families. Parental psychological distress was measured using the 6-item Kessler Psychological Distress Scale (K6) at offspring ages 3, 5, 7, and 11 years. At age 14 years, the child’s depressive symptoms were measured with the Short Mood and Feelings Questionnaire (SMFQ). Parallel process latent growth curves examined unfolding of maternal and paternal psychological distress symptoms and assessed whether growth parameters (intercepts and slopes) of each parent’s trajectory predicted adolescent SMFQ scores.

**Results:**

Intercepts and slopes of maternal and paternal symptom trajectories were positively correlated, indicating parallel development. The maternal and paternal intercepts were independently predictive of adolescent SMFQ scores, as was the maternal, but not the paternal, slope after adjustment for confounding.

**Conclusion:**

Maternal and paternal psychological distress symptoms develop in parallel from early to late childhood. Higher levels of psychological distress experienced by either parent in the early years and increasing levels of maternal symptoms across childhood predict higher levels of offspring depression in adolescence. These findings highlight the importance of early intervention targeting psychological distress of both parents of young children.

## Plain language summary

Using data from 8,888 families in the United Kingdom Millennium Cohort Study, trajectories of parental psychological distress trajectories when their child was 3 to 11 years of age were modeled and then linked to offspring depressive symptoms at age 14. Distress levels in mothers and fathers developed in parallel across their child’s early years, indicating tightly aligned parental mental-health patterns. Higher psychological distress in either parent during their child’s early years, and a steady increase in maternal distress, were associated with more pronounced adolescent depressive symptoms. These findings underscore the value of sustained mental-health support for both parents during their child’s early years to help reduce later risk for their children.

To date, studies investigating the influence of parental mental health on adolescent mental health have largely focused on maternal, rather than paternal, symptoms.^1^^,^^2^ Nonetheless, it seems pertinent to advance our understanding of how the mental health trajectory of each parent may impact adolescent mental health, given that fathers have become increasingly involved in child-rearing.^3^^,^^4^ Furthermore, little is known about the codevelopment of maternal and paternal mental health within families. If both paternal and maternal depressive symptoms, for example, have an effect on depressive symptoms in offspring and develop in parallel, it is unlikely that one parent could take a compensatory role and counteract the effects on children of the other parent’s mental health difficulties. Therefore, information about the nature of development of maternal and paternal mental health difficulties across the childhood years could be crucial in preventing offspring mental health difficulties in adolescence. The present study aimed to further our understanding of how psychological distress experienced by each parent across the childhood years can influence the level of depressive symptoms of offspring in adolescence.

Longitudinal investigations and meta-analytic reviews have demonstrated lasting effects of maternal depression on psychopathology of offspring,^5^^,^^6^ including depression, via several mechanisms. First, maternal depression during pregnancy may contribute to an adverse intrauterine environment that can affect fetal brain development, thus increasing the risk for mental health problems via epigenetic processes.^7^^,^^8^ Genes can also play a role directly, as offspring of parents with depression are shown to inherit a genetic predisposition to depression.^9^ Also, a multitude of environmental and social factors may explain the transmission of parents’ mental ill-health to offspring, including poverty,^10^ stress,^11^ and social isolation.^12^ Additionally, parental depression may influence parenting behaviors^13^ that can affect the child’s long-term socioemotional development.^14^

In contrast, the long-term influence of paternal psychological distress on psychological outcomes in developing offspring has received much less attention.^15^, ^16^, ^17^ Most of the available longitudinal evidence on this topic concerns patterns in infancy and the childhood years.^16^^,^^18^, ^19^, ^20^, ^21^, ^22^ There is much less evidence regarding effects in adolescence, but the existing data suggest that paternal psychological distress in childhood is associated with the experience of depression in offspring in adolescence, independently of, and similarly to, maternal psychological distress.^16^ Lewis *et al.*^16^ also showed that adolescents are at greater risk of developing depression if both parents are depressed, perhaps reflecting greater genetic vulnerability, increased exposure to environmental risk factors, or both. This finding is particularly important in light of evidence for assortative mating for affective disorders.^23^

Another shortcoming in the available literature is the typically cross-sectional measurement of parental symptoms, which means that the dynamic and potentially diverse unfolding of parental symptoms over time cannot be captured. However, affective symptoms are not stable traits. Longitudinal studies have demonstrated that individuals in general population samples show diverse developmental trajectories of affective problems across life stages.^24^^,^^25^ Still, only a handful of studies have looked at the prospective associations of parental trajectories of depressive symptoms with levels of depression in offspring, and no study to date has modeled maternal and paternal depressive symptom trajectories in parallel to disentangle any unique contributions. Studies that have modeled maternal affective symptoms prospectively in relation to long-term psychological outcomes of offspring suggest that increasing levels of maternal depression may be associated with problem behaviors in adolescence,^24^ albeit the evidence remains mixed.^26^

Overall, very little is known about whether trajectories of maternal and paternal psychological distress develop in parallel, how changes in distress levels of fathers over time might impact depressive symptoms in adolescents, and what the independent contribution of maternal and paternal distress trajectories is to adolescent depression. The present study sought to address these gaps in the literature using data from the largest and most recent birth cohort in the United Kingdom, the Millennium Cohort Study (MCS). It was hypothesized that trajectories of maternal and paternal psychological distress symptoms will develop in parallel due to assortative mating and shared environmental influences (although we did not directly test for assortative mating, we explored whether it could be suggested by the dynamics of such a parallel unfolding of symptoms, akin to those documented for affective disorders)^23^ and elevated or increasing levels of psychological distress in a father and mother will be independently predictive of elevated symptoms of depression in their adolescent.

## Method

Data for this study came from the first 6 waves (sweeps) of MCS, a large, population-based, prospective cohort study following children born in the United Kingdom in 2000-2002. Families were first assessed when the children were 9 months old (MCS1) and subsequently at ages 3 (MCS2), 5 (MCS3), 7 (MCS4), 11 (MCS5), and 14 (MCS6). The number of participating families in each sweep was 18,522, 15,590, 15,246, 13,857, 13,287, and 11,714, respectively.^27^ Parents self-reported their psychological distress from sweep 2 onward. Cohort members self-reported their depressive symptoms during sweep 6, when they were 14 years old. Ethical approval was obtained from National Health Service Multi-Centre Ethics Committees. Parents gave informed consent before interviews took place, and children gave assent at age 11 and consent at age 14.

### Participants

The analytic sample of the study was restricted to children (singletons and first-born twins or triplets) with complete data on the outcome measure, whose parents also had valid data on psychological distress symptoms in at least one of sweeps 2 (age 3; when their symptoms were first measured) to 5 (age 11). The sample included children who cohabited with either their biological father or a stepfather during at least one sweep (between ages 3 and 11). Children from continuously single-parent families were excluded. The resulting sample consisted of 8,888 children and their parents.

### Assessment of Depressive Symptoms

Adolescent depressive symptoms were measured using the Short Mood and Feelings Questionnaire (SMFQ) at age 14.^28^ The SMFQ is a 13-item self-report measure of depressive symptoms that was developed for use among children and adolescents 6 to 17 years old in the general population. Total scores range from 0 to 26, with higher scores indicating more depressive symptoms. The variable was used as a continuous variable in our study, albeit a cutoff score of 8 or higher has been shown to yield high sensitivity and specificity rates for major depression. Reliability of the SMFQ was high, as indicated by a Cronbach's α of .93 in the sample.

Maternal and paternal depressive (psychological distress) symptoms were measured using the 6-item Kessler Psychological Distress Scale (K6) in sweeps 2, 3, 4, and 5. The K6 is a validated self-report instrument that was developed to estimate psychological distress in general population samples. Total scores range from 0 to 24, with higher values indicating more difficulties. K6 scores of 0 to 7 indicate low risk, 8 to 12 indicate moderate risk, and 13 to 24 indicate high risk of psychological distress.^29^ Cronbach's α values for maternal K6 were .84, .86, .87, and .89 across sweeps 2 to 5, respectively. For fathers, Cronbach's α values were .79, .81, .82, and .85, respectively.

### Covariates

Covariates were individual and family characteristics that previous evidence has associated with both parental and adolescent depression. Individual characteristics comprised the sex (male/female) and ethnicity of the cohort member. Ethnicity, originally captured by a 6-, 8-, and 11-category census classification in MCS, was dummy-coded as minority ethnic or White because minority ethnic groups were represented by very few cases, not allowing for sufficient statistical power in comparative analyses. Family characteristics included paternal and maternal educational level (university degree obtained or not) and socioeconomic disadvantage (above/below 60% of the median equivalized disposable income of the population, according to Organisation for Economic Cooperation and Development data). Arrival of new sibling (yes/no) between MCS2 and MCS5 was controlled for, to account for the potential effects of postnatal depression on parents. Changes in family structure (entries and exits of fathers and mothers) and biological status of father (yes/no) were also controlled for across sweeps MCS2 to MCS5.

### Statistical Analyses

**Figure 1:**
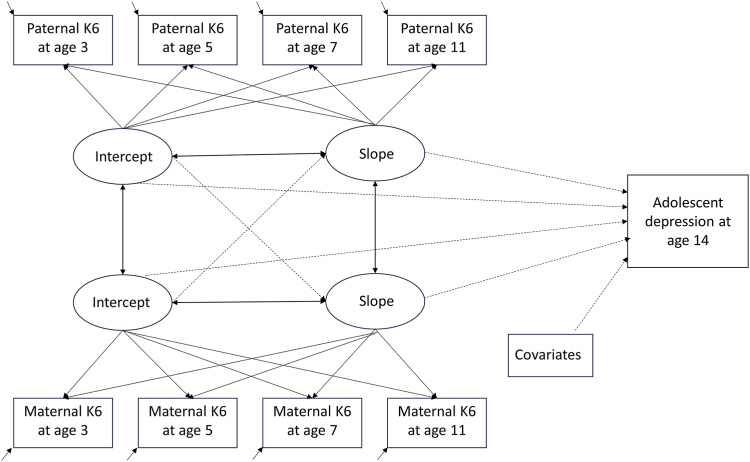
Specified Parallel Process Latent Growth Curve Model for Maternal and Paternal Depressive Symptoms at Child’s Ages 3 to 11 Including Adolescent Depression at Age 14 as a Distal Outcome ***Note:****Observed variables are enclosed by rectangles. Latent variables are enclosed by ellipses. Single-headed arrows signify regression paths. Double-headed arrows signify correlations. K6 = 6-item Kessler Psychological Distress Scalescore.*

Parallel process latent growth curve modeling (LGCM) was used to describe the unfolding of maternal and paternal depressive symptoms simultaneously from when the child was ages 3 to 11. LGCM estimates the growth of developmental trajectories, and parallel process LGCM allows the modeling of 2 growth trajectories simultaneously. This facilitates the evaluation of the growth parameters of separate trajectories (in this case, the psychological distress of the mother and father) and the relations between them, for example, whether one parent’s rate of change in psychological distress symptoms correlates with that of the other parent. LGCM also allows for the incorporation of outcome variables, i.e., the growth parameters can be tested as predictors of an outcome. This enabled us to test whether growth parameters of the mother and father (baseline K6 scores, scores when their child was age 3, and rates of change in symptom development at ages 3-11 years) independently predict their child’s depressive symptoms in adolescence at age 14 years. Although LGCM can also model nonlinear trajectories, we examined linear trajectory models for parental K6 trajectories based on several considerations, including model parsimony, simplicity of interpretation, and lack of strong theory for nonlinear change in this population’s symptom development. Figure 1 illustrates our parallel process latent growth curve model.

We ran a series of models. First, we fitted a parallel process model without including the outcome variable or confounders to assess the codevelopment of paternal and maternal psychological distress (depressive symptoms) trajectories. Next, we ran a parallel process model to estimate the predictive ability of each of the paternal and maternal psychological distress trajectories for depression levels of adolescent offspring before (model A) and after (model B) adjustment for confounding. Finally, we ran a supplementary analysis (Supplement 1, available online) to test whether depressive symptoms of mothers and fathers have the same impact on adolescents of the same and opposite sex. After stratifying the sample by offspring sex, we ran a parallel process model with and without adjustment for confounding. For all models, we estimated correlations between intercepts and slopes within a parent. This allowed us to assess whether the average psychological distress score at baseline (intercept) and the rate of change in scores over time (slope) are associated within a parent. Between parents, we estimated correlations between intercepts and between slopes to assess to what extent initial levels of symptoms and their development over time are correlated between parents. We also estimated regression paths from the intercept of one trajectory to the slope of the other to assess whether the initial level of one parent’s symptoms was associated with the other parent’s course of symptoms over time (Figure 1).

All models were run using the maximum likelihood with robust standard errors estimator to account for the skewed distribution of the data. Study-specific clustering, stratification, and weight variables were included in the analysis to control for attrition, nonresponse, and the disproportionate oversampling of disadvantaged areas in MCS. Missing data were handled using full information maximum likelihood, which capitalizes on all available data to estimate parameters and is considered superior to more traditional methods used to treat missing data, such as multiple imputation.^30^ The fit of each model was assessed using criteria proposed by Hu and Bentler^31^; good fit was considered as comparative fit index (CFI) ≥0.95, Tucker-Lewis index (TLI) ≥0.95, root mean square error of approximation (RMSEA) <0.06, and standardized root mean square residual (SRMR) <0.08. No restrictions were imposed on the residuals of the growth curve indicators, the latent growth parameters, or the means or variances for any of the observed covariates. All analyses were conducted using fixed-time scores, centered at the first time point. Specifically, time scores of 0, 1, 2, and 4 were assigned for the K6 assessments corresponding to offspring ages 3, 5, 7, and 11 years, respectively. All models were run in Mplus v7.4.^32^

## Results

The characteristics of the analytic sample and percentage of missing data across variables are summarized in Table 1. The children in the analytic sample (N = 8,888, 50.5% female) were predominantly White (83.6%), and most families lived above the poverty line (73.9%). Most children (90.8%) lived with both biological parents during at least one of the sweeps across ages 3 to 11 years.

**Table 1:** Descriptive Statistics of Analytic Sample (Unweighted Data)

	N	Mean	(SD)	Missing data (%)
Maternal psychological distress (K6 scores)				
Child’s age 3	7,746	2.99	(3.42)	12.9
Child’s age 5	7,978	2.86	(3.49)	10.2
Child’s age 7	7,881	2.83	(3.53)	11.3
Child’s age 11	8,082	3.62	(4.02)	9.1
Paternal psychological distress (K6 scores)				
Child’s age 3	6,874	2.85	(3.04)	22.6
Child’s age 5	6,937	2.93	(3.24)	22.0
Child’s age 7	6,535	2.89	(3.28)	26.5
Child’s age 11	6,768	3.76	(3.79)	23.9
Adolescent (age 14) depression (SMFQ scores)	8,888	4.07	(4.00)	0.0
	**N**	**(%)**		**Missing data (%)**
Child’s sex				0.0
Male	4,402	(49.5)		
Female	4,486	(50.5)		
Child’s ethnicity				0.6
Minority ethnic	1,404	(15.8)		
White	7,431	(83.6)		
Socioeconomic status				4.5
OECD above 60% median	6,571	(73.9)		
OECD below 60% median	1,917	(21.0)		
Paternal education				14.2
University degree	4,529	(51.0)		
No university degree	3,094	(34.8)		
Maternal education				4.4
University degree	3,442	(38.7)		
No university degree	5,051	(56.8)		
Change in family structure				9.9
No change in family structure	6,054	(68.1)		
Change in family structure	1,950	(21.9)		
Arrival of a new sibling				11.3
No new sibling(s) born between MCS2 and MCS5	6,532	(73.5)		
New sibling(s) born between MCS2 and MCS5	1,351	(15.2)		
Status of father				1.8
Biological father	8,070	(90.8)		
Stepfather	661	(7.4)		

Note: K6 = 6-item Kessler Psychological Distress Scale; MCS = Millennium Cohort Study; OECD = Organisation for Economic Cooperation and Development; SMFQ = Short Mood and Feelings Questionnaire.

### Parallel Process LGCM for Paternal and Maternal Psychological Distress Trajectories

A parallel process latent growth curve model was run to assess the relation between paternal and maternal growth parameters of psychological distress trajectories before including the outcome variable or confounders. The model showed excellent fit to the data (CFI = 0.97, TLI = 0.96, RMSEA = 0.03, SRMR = 0.03). Table 2 shows the mean growth parameter estimates for maternal and paternal depressive symptoms. The slope estimates show that, on average, both maternal and paternal trajectories were characterized by small, yet statistically significant, increases between MCS2 and MCS5.

**Table 2:** Growth Parameter Estimates of Parallel Process Latent Growth Curves for Maternal and Paternal Psychological Distress Symptoms

	Estimate	(SE)	*p*
Intercept			
Mothers	2.98	(0.05)	<.001
Fathers	2.83	(0.04)	<.001
Slope			
Mothers	0.19	(0.01)	<.001
Fathers	0.27	(0.02)	<.001
Intercept–slope correlations			
Maternal intercept and paternal intercept	0.28	(0.03)	<.001
Maternal slope and paternal slope	0.52	(0.11)	<.001
Maternal intercept and maternal slope	0.03	(0.08)	.66
Paternal intercept and paternal slope	−0.04	(0.07)	.57
Unstandardized regression paths			
Maternal slope on paternal intercept	−0.02	(0.01)	.09
Paternal slope on maternal intercept	0.01	(0.01)	.33

**Figure 2:**
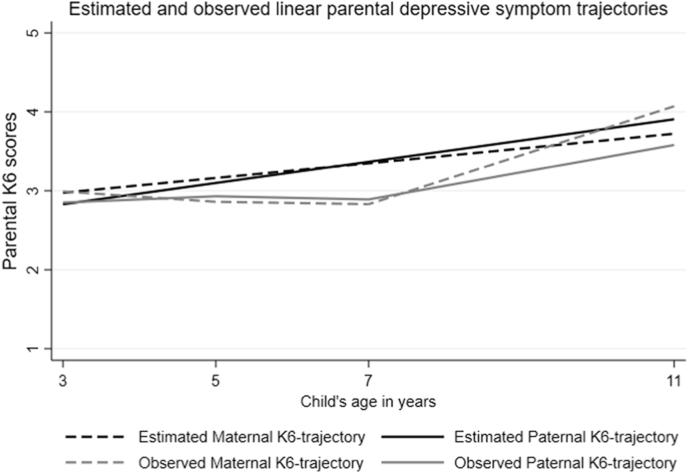
Observed and Estimated Trajectories of Maternal and Paternal Depressive Symptoms ***Note:****K6 = 6-item Kessler Psychological Distress Scale (score).*

There was a significant association between the intercepts (baseline scores) of trajectories of mothers and fathers (*r* = 0.28, *p* < .001), signifying that mothers who reported higher levels of psychological distress when their children were 3 were likely to co-parent with fathers who also had higher K6 scores. A positive connection was also found between the slopes of the trajectories of maternal and paternal psychological distress (*r* = -.52, *p* = .001), indicating that, to an extent, the 2 trajectories develop in parallel. Figure 2 shows the parallel unfolding of maternal and paternal depressive symptoms from when the cohort members were 3 through 11 years of age. Visual inspection of the data suggested that the observed symptom means over the 4 time points can be described by linear trajectories, but that they could also be captured using a nonlinear quadratic term (reflected in Figure 2, which illustrates both observed and estimated means). As a robustness check we compared the estimated values for both maternal and paternal trajectories before and after including a quadratic slope in the model. We found that the estimated means of linear and nonlinear trajectories were closely aligned, thus lending support to our decision to proceed with the more parsimonious linear trajectories.

### Predictive Ability of Parental Psychological Distress Trajectories for Offspring Depression

Table 3 presents the results of 2 latent growth curve models examining the connection between the growth parameters of maternal and paternal depressive symptoms and their relation to depressive symptoms experienced by offspring during adolescence. The first parallel process latent growth curve model (model A) was run without adjustment for covariates. It showed excellent fit to the data (CFI = 0.97, TLI = 0.96, RMSEA = 0.03, SRMR = 0.03). The second model (model B) included covariates and showed excellent fit to the data as well (CFI = 0.97, TLI = 0.95, RMSEA = 0.02, SRMR = 0.02).

**Table 3:** Unstandardized Unadjusted (Model A) and Adjusted (Model B) Regression Coefficients of the Relation Between Growth Parameters of Maternal and Paternal Psychological Distress Trajectories and Adolescent’s Depression Scores

	Model A			Model B		
	Coefficient	(SE)	*p*	Coefficient	(SE)	*p*
Parental psychological distress growth parameters						
Maternal intercept	0.20	(0.05)	<.001	0.18	(0.04)	<.001
Maternal slope	2.13	(0.85)	.01	1.93	(0.69)	.005
Paternal intercept	0.13	(0.06)	.04	0.12	(0.05)	.03
Paternal slope	−0.55	(0.62)	.38	−0.39	(0.51)	.44
Covariates						
Sex, female	—		—	3.04	(0.16)	<.001
Ethnicity, minority ethnic	—		—	−0.71	(0.25)	.004
Socioeconomic status, OECD below 60% median	—		—	0.18	(0.24)	.45
Paternal education, university degree or higher	—		—	−0.15	(0.17)	.38
Maternal education, university degree or higher	—		—	0.10	(0.17)	.55
Change in family structure	—		—	0.54	(0.22)	.02
Arrival of new sibling	—		—	0.44	(0.24)	.07
Status of father, biological father	—		—	−0.78	(0.38)	.04

Note: The associations presented represent the direct effects of the confounders on adolescent depression after accounting for the growth parameters of the parental psychological distress trajectories. Growth parameters may in fact serve as mediators in the causal pathway between confounders and the outcome. All regression coefficients of growth parameters presented are adjusted for one another. Some growth parameters (eg, the slopes) could in fact also act as mediators for the relation between remaining growth parameters and the outcome. OECD = Organisation for Economic Cooperation and Development.

Before adjusting for covariates (model A), the growth parameters (intercept and slope) of the maternal psychological distress trajectory were positively associated with adolescent depressive symptoms. The intercept of paternal symptoms was also significantly associated with adolescent symptoms, whereas paternal slope was not. After adjusting for covariates (model B), the associations noted in model A were attenuated yet remained significant. Specifically, higher values in the maternal intercept (β = .18, SE = 0.04, *p* < .001), maternal slope (β = 1.93, SE = 0.69, *p* = .005), and paternal intercept (β = .12, SE = 0.05, *p* = .03) were independently predictive of higher SMFQ scores in adolescent offspring. Sex, ethnicity, change in family structure, and father’s biological status were also independently predictive of SMFQ scores, whereas household income, maternal education, paternal education, and arrival of additional siblings were not. This suggests that female offspring (β = 3.04, SE = 0.16, *p* < .001), White offspring (β = −.71, SE = 0.25, *p* = .004), offspring who experienced a change in family structure (β = .54, SE = 0.22, *p* = .02), and offspring living with nonbiological fathers (β = −.78, SE = 0.38, *p* = .04) were more likely to have higher SMFQ scores in adolescence. Results of the sex-stratified analyses estimating the impact of parental psychological distress on the depressive symptoms of same- and opposite-sex offspring are presented in Supplement 1, available online.

## Discussion

The results indicate that among 2-parent families with young and primary school children in the United Kingdom, the trajectories of maternal and paternal psychological distress (depressive symptoms) develop in parallel. Specifically, both mothers and fathers experienced an increase in psychological distress from the time their children were 3 through 11 years old, albeit their scores remained within the low-risk category for psychological distress.^29^ Importantly, both maternal and paternal depressive symptoms, measured when children were age 3, were independently predictive of depressive symptom scores of children when they were age 14, suggesting that exposure to higher levels of both maternal and paternal depressive symptoms in early childhood can have detrimental long-term effects. Moreover, offspring who were exposed to a trajectory of increasing maternal psychological distress across their childhood years were more likely to have higher levels of depressive symptoms at age 14. Sex-stratified analyses additionally suggested that the effects of maternal psychological distress on the risk of depression for boys, rather than girls, was the main driver of the significant associations, albeit the effects of each of the maternal and paternal growth parameters on adolescent depression were comparable in size between sexes.

### Trajectories of Maternal and Paternal Psychological Distress Develop in Parallel

The correlation between the intercepts of maternal and paternal symptom trajectories indicated that mothers were likely to co-parent with fathers with similar levels of psychological distress. Importantly, a relatively strong and statistically significant correlation was also found between the slopes of the 2 trajectories (*r* = 0.52, *p* < .01), indicating that the trajectories of maternal and paternal depressive symptoms develop in parallel. Together, these findings are consistent with existing research on assortative mating, which suggests that individuals typically choose mates who are similar to them.^23^^,^^33^ Another possible explanation may be that, as partners, the 2 parents share exposures likely to be depressogenic, such as adverse life events or stressful experiences. A third explanation, however, may be that the trajectories appear to be developing in parallel but may in fact be lagging, with one partner’s elevated symptoms increasing the symptoms of the other partner.^34^ This in turn suggests that, without intervention, psychological distress in one parent can cause symptoms in the other parent, creating a spiral of deteriorating mental health in both. Nonetheless, the regression coefficients linking the intercept of one parent’s trajectory with the slope of the other parent were not statistically significant in our study, suggesting absence of reciprocal influences between parental psychological distress trajectories.

As mentioned above, a reason why parental trajectories of psychological distress develop in parallel may be because parents share and create environmental stressors. The child is a central part of the parents’ shared environment, and child problems may be a common stressor for both parents.^35^^,^^36^ It is possible, for instance, that child behavior problems escalate parental stress and increase interparental conflict, which is, in turn, associated with depressive symptoms in both parents.^37^ It is important, however, to note that the severity of parental depressive symptoms in this study was not clinically significant. Psychological distress trajectory scores were within the low-risk category of psychological distress (≤7) across assessment waves. This finding is crucial as it suggests that even modestly elevated levels of psychological distress in the early years can have a significant impact on the risk of offspring depression in adolescence. In parallel, the finding that even subtle increases in depressive symptoms in mothers across the childhood years can also raise this risk underscores the idea that even low levels of chronic maternal psychological distress may impair mental well-being of offspring in adolescence. The implications of this finding are significant. Assortative mating for affective disorders may place some children at greater risk of inheriting genetic vulnerability for depression from 2 depressed parents. Having one parent without depression has been shown to partially moderate the effects of the other parent’s depression.^38^ The parent without depression can provide support for the parent with depression, a healthy role model for the child, and parenting that promotes healthy emotional development.^39^^,^^40^ However, a child’s risk of developing depression is compounded if increasing depressive symptoms in one parent increase symptoms in the other. Together, these findings suggest that interventions seeking to prevent depression in adolescents should screen for and address psychological distress in both parents as early as the preschool years, as well as chronic depressive symptoms, even mild, in mothers. According to our findings, such interventions can be beneficial in preventing elevated depressive symptom scores in adolescents even if parental symptoms are below the threshold that would deem them clinically significant. Nonetheless, it is worth repeating that the K6, used here, measures psychological distress rather than providing a diagnostic assessment of depression. Future research could expand our findings by incorporating comprehensive diagnostic tools.

### Impact of Paternal Psychological Distress Over Time on Adolescent Depression

The finding that children who were exposed to higher levels of paternal depressive symptoms when they were 3 years old were more likely to have higher levels of depressive symptoms in adolescence is particularly salient. It adds to the growing body of research demonstrating that depressive symptoms in fathers are predictive of depression in adolescent offspring, independently of symptoms in mothers,^16^ via several mechanisms including parenting.^41^, ^42^, ^43^ Therefore, a trajectory of increasing paternal distress was also expected to influence the risk of depression in adolescence. However, the present study found that although paternal depressive symptoms increased from when children were ages 3 to 11, the increase was not associated with higher levels of depressive symptoms in adolescents.

The finding that the intercept, but not the slope, of the paternal symptom trajectory predicted adolescent depression could be indicative of shared genetic risk. Drawing definitive conclusions about heritability is not possible as the study did not use genetic data. However, because the majority of children within the sample lived with both biological parents, this assumption is likely to hold. It may also reflect the importance of timing effects for such parental inputs. For example, Connell and Goodman^44^ found that maternal depression has greater effects on child internalizing symptoms than paternal depression during early and mid-to-late childhood, whereas in children older than 13, the effects of paternal depression surpass those of maternal depression. One plausible explanation is that younger children are predominantly cared for by mothers, whereas fathers become more involved in parenting as children grow older. This would, in turn, explain the association between paternal and offspring depression that has been documented in studies with older participants (ages 11-15).^17^^,^^45^^,^^46^ Future studies could explore if paternal symptom trajectories from early childhood to middle adolescence and beyond yield different results than we observed here.

An alternative interpretation of the findings is that the influence of depression in either parent on children is strongest early in life, a critical developmental window. This would explain why both the paternal and the maternal intercept were predictive of adolescent SMFQ scores. Sweeney and MacBeth^47^ conducted a systematic review of studies examining the association between paternal depression and child outcomes, with participants from 1 month to 21 years old. They reported that the association between paternal depression and child internalizing problems was strongest in samples of younger children. Most studies included in the meta-analysis by Connell and Goodman^44^ were published between 1970 and 2001, whereas most of those reviewed by Sweeny and MacBeth^47^ were published between 2000 and 2014. It is plausible that involvement of fathers in caring for infants and younger children increased, as a proportion of total parental childcare, between the 2 studies. Statutory paternity leave was introduced in the United Kingdom in 2003, and the percentage of fathers with access to flexi-time increased from 22% to 54% between 2001 and 2005.^48^ Importantly, data collection for the second sweep of the MCS coincided with this significant societal change. Depressive symptoms experienced by fathers were first measured when their children were 3 years old, in 2004. Perhaps the fathers in our sample were more involved in caring for younger children than fathers in earlier studies. Therefore, the association between paternal and offspring depression in the last 20 years in the United Kingdom may be more similar to the association between maternal and offspring depression than previously thought. Our study clearly suggests that early exposure to depressive symptoms in either parent is a risk factor for depression in adolescence.

### Impact of Maternal Psychological Distress Over Time on Adolescent Depression

As mothers often act as the primary caregiver, and parenting is impaired by depression and shapes offspring outcomes,^13^^,^^14^ it is not surprising that exposures to maternal depressive symptoms at age 3 and across childhood were associated with adolescent depressive symptoms. This finding builds on previous evidence suggesting that increasing levels of depression in mothers are associated with adverse outcomes in adolescence.^24^ Importantly, it also shows that such associations hold even after adjustment for the depressive symptom trajectory of fathers. However, the results of the supplementary analysis suggest that the main effects reported were driven mainly by the negative impact of maternal depression (at age 3 and across childhood) on male offspring. This finding is consistent with existing research that suggests boys are more vulnerable to the effects of maternal postnatal depression than girls.^49^ It is, however, noteworthy that the magnitude of the relations was not significantly different between girls and boys. It is thus likely that the effect sizes are comparable, and any differences would be detectable only in larger samples.

Our study has several strengths. It was the first to model the parallel development of maternal and paternal psychological distress symptoms and relate it to offspring risk of depression in adolescence. The use of state-of-the-art analysis, a large sample representative of the UK population, and data from 5 time points across a 10-year period enabled us to provide a detailed, longitudinal insight into the dynamic associations between both paternal and maternal psychological distress and adolescent depressive symptoms. This study also adopted a methodologically innovative approach that demonstrated that compared with the effect of early maternal psychological distress, the effect of early paternal psychological distress on adolescent mental health may be smaller but still highly significant. Importantly, the effects of both paternal and maternal psychological distress were statistically significant despite parent K6 scores falling in the low-risk category for psychological distress.^29^ This, in conjunction with the finding about the effect of the slope of the maternal symptom trajectory, suggests that even subtle increases in maternal psychological distress can have a long-lasting impact on offspring risk of depression. Moreover, our study highlights the added value of modeling trajectories of parental psychological distress instead of relying on cross-sectional assessments. This approach enabled the identification of the positive slope of maternal psychological distress trajectory as a significant predictor of offspring risk of depression in adolescence, even in the presence of low maternal K6 scores.

Nevertheless, our findings may have limited generalizability for adolescents from ethnic minority backgrounds and can be applied only to adolescents growing up in 2-parent families. We also acknowledge that some of the confounders modeled as having a direct impact on the outcome only, such as changes in the family structure, may in fact lie on the causal pathway between parental and offspring depression. Future research could delve deeper into the nuanced dynamics of such covariates and their impact on the mental health of both parents and offspring. It is also worth noting that the potentially salient impact of psychological distress of noncustodial parents on adolescent depressive symptoms could not be considered in our analyses, as MCS did not follow up these parents. Furthermore, our study did not use molecular genetic information, and hence it was not possible to determine the extent of genetic and environmental influence in the links identified. Lastly, it was not possible to model trajectories of parental psychological distress from infancy. During the first sweep of the MCS, when cohort members were 9 months old, parental psychological distress was measured with another instrument (the Malaise Inventory). Consequently, to avoid introducing bias by using 2 different instruments, we modeled trajectories only from age 3.

In conclusion, this is the first study to track the course of psychological distress in both parents across 4 time points in almost a decade and relate it to depressive symptoms experienced by adolescent offspring. The results suggested that psychological distress trajectories of parents during their child’s preschool and primary school years develop in parallel. Moreover, offspring who were exposed to a trajectory of increasing maternal psychological distress across childhood had higher levels of depressive symptoms in adolescence. Both maternal and paternal psychological distress symptoms experienced when children were very young were independently predictive of depressive symptoms in adolescence, suggesting that exposure to higher levels of either maternal or paternal depressive symptoms in early childhood has long-term detrimental effects. These novel findings have significant implications for policymakers and practitioners, suggesting that it is important to target psychological distress in both parents, especially during early childhood of their offspring.

## CRediT authorship contribution statement

**Katia Mace:** Writing – original draft, Formal analysis. **Maria Sifaki:** Writing – review & editing, Formal analysis. **Emily Midouhas:** Writing – review & editing, Conceptualization. **Eirini Flouri:** Writing – review & editing. **Efstathios Papachristou:** Writing – review & editing, Supervision, Formal analysis.
