# Supplementary material for: The Course of Parental Psychological Distress in Childhood and Adolescent Depression
Source: JAACAP Open. 2024 Jul 8;3(3):634–44. doi: 10.1016/j.jaacop.2024.05.003 (PMC12414316; doi:10.1016/j.jaacop.2024.05.003)
Supplement: Supplement 1 [file mmc1.docx]

***Supplement 1***

As a supplementary analysis, sex-stratified models were run, to test whether mother’s and father’s depression have the same impact on children of the same and opposite sex, before and after adjustments for covariates. The results of these models are summarised in Table S1. We found that maternal, but not paternal, intercept (*β*=.16, *p*<.001) and slope (*β*=1.22. *p* = .010) were predictive of SMFQ scores among male offspring, in the unadjusted model. Maternal intercept (*β*=.14*, p*=.002) and slope (*β*=1.23, *p*=.006) continued to be predictive of boys’ SMFQ scores in the adjusted model. Neither maternal nor paternal growth parameters were predictive of SMFQ scores among female offspring. Nonetheless, pairwise comparisons for the regression coefficients of the paths linking the growth parameters with SMFQ scores in the adjusted models revealed that the magnitude of the estimates was not significantly different between sexes. P-values of Wald tests of parameter constraints for these comparisons ranged from .41 to .62. Specifically, the results of the comparisons between the regression estimates of each growth parameter (maternal and paternal intercepts and slopes) on male and female adolescents’ SMFQ scores were: a) χ^2^ (1)= .25, p= .62 for maternal intercept; b) χ^2^ (1)= .41, p= .52 for maternal slope; c) χ^2^ (1)= .69, p= .41 for paternal intercept; d) χ^2^ (1)= .47, p= .50 for paternal slope.

| **Table S1**. Unstandardised unadjusted (Model A) and adjusted (Model B) regression coefficients for the relationship between growth parameters of maternal and paternal psychological distress trajectories on male and female adolescent offspring’s depression scores. | | | | | | | | |
| --- | --- | --- | --- | --- | --- | --- | --- | --- |
|  | *Model A* | | *Model B* | | *Model A* | | *Model B* | |
|  | *Male offspring (N=4402)* | | | | *Female offspring (N=4486)* | | | |
|  | *Coeff. (SE)* | *p-value* | *Coeff. (SE)* | *p-value* | *Coeff. (SE)* | *p-value* | *Coeff. (SE)* | *p-value* |
| Parental psychological distress growth parameters  Maternal intercept  Maternal slope  Paternal intercept  Paternal slope | .16 (.05)  1.22 (.48)  .07 (.05)  .03 (.53) | <.001  .01  .11  .95 | .14 (.05)  1.23 (.45)  .06 (.05)  .07 (.49) | .002  .006  .17  .89 | .18 (.23)  6.38 (10.30)  .34 (.45)  -2.62 (5.19) | .43  .54  .45  .61 | .20 (.11)  4.49 (5.06)  .23 (.20)  -1.58 (2.42) | .06  .38  .25  .51 |
| Covariates  Ethnicity, non-White  Socioeconomic status, OECD below 60% median  Paternal education, non-University educated  Maternal education, non-University educated  Change in family structure  Arrival of new sibling  Status of father, step-father | --- | --- | -.18 (.30)  -.10 (.27)  .24 (.22)  -.04 (.21)  .59 (.26)  .24 (.28)  -.64 (.49) | .54  .70  .28  .85  .02  .40  .19 | --- | --- | -1.29 (.38)  .52 (.40)  -0.53 (.26)  .23 (.27)  .53 (.37)  .59 (.37)  -.92 (.61) | .001  .19  .04  .39  .11  .11  .13 |
| ***Note****: a) The associations presented represent the direct effects of the confounders on adolescent depression after accounting for the growth parameters of the parental psychological distress trajectories. Growth parameters may in fact serve as mediators in the causal pathway between confounders and the outcome; b) all regression coefficients of growth parameters presented are adjusted for one another. Some growth parameters (e.g., the slopes) could in fact also act as mediators for the relationship between the remaining growth parameters and the outcome.* | | | | | | | | |
